# VanZ Reduces the Binding of Lipoglycopeptide Antibiotics to *Staphylococcus aureus* and *Streptococcus pneumoniae* Cells

**DOI:** 10.3389/fmicb.2020.00566

**Published:** 2020-04-03

**Authors:** Vladimir Vimberg, Leona Zieglerová, Karolína Buriánková, Pavel Branny, Gabriela Balíková Novotná

**Affiliations:** ^1^Laboratory for Biology of Secondary Metabolism, Institute of Microbiology of the Czech Academy of Sciences, Prague, Czechia; ^2^Laboratory of Cell Signaling, Institute of Microbiology of the Czech Academy of Sciences, Prague, Czechia

**Keywords:** *Staphylococcus aureus*, *Streptococcus pneumoniae*, antibiotic resistance, lipoglycopeptide antibiotics, VanZ

## Abstract

*vanZ*, a member of the *VanA* glycopeptide resistance gene cluster, confers resistance to lipoglycopeptide antibiotics independent of cell wall precursor modification by the *vanHAX* genes. Orthologs of *vanZ* are present in the genomes of many clinically relevant bacteria, including *Enterococcus faecium* and *Streptococcus pneumoniae*; however, *vanZ* genes are absent in *Staphylococcus aureus*. Here, we show that the expression of enterococcal *vanZ* paralogs in *S. aureus* increases the minimal inhibitory concentrations of lipoglycopeptide antibiotics teicoplanin, dalbavancin, oritavancin and new teicoplanin pseudoaglycone derivatives. The reduction in the binding of fluorescently labeled teicoplanin to the cells suggests the mechanism of VanZ-mediated resistance. In addition, using a genomic *vanZ* gene knockout mutant of *S. pneumoniae*, we have shown that the ability of VanZ proteins to compromise the activity of lipoglycopeptide antibiotics by reducing their binding is a more general feature of VanZ-superfamily proteins.

## Introduction

Glycopeptide antibiotics are important for the treatment of multidrug-resistant infections caused by gram-positive bacteria. The emergence and spread of enterococcal strains resistant to vancomycin and teicoplanin (VRE) is a serious public health concern (Uttley et al., 1989). In VRE-resistant strains, cell wall biosynthesis is reprogramed to produce peptidoglycan precursors containing either D-alanine-D-lactate (D-Ala-D-Lac) or D-alanine-D-serine instead of the dipeptide D-alanine-D-alanine (D-Ala-D-Ala). As a result, the affinity of glycopeptide antibiotics to peptidoglycan dramatically decreases (Bugg et al., 1991; Lessard and Walsh, 1999). The three essential enzymes responsible for the precursor modification are encoded in *vanHAX* gene clusters. In the *vanA*-type gene cluster, two additional genes, *vanY* and *vanZ*, also contribute to glycopeptide resistance. VanY, a D, D-carboxypeptidase, eliminates D-Ala-D-Ala from peptidoglycan precursors, minimizing the number of primary binding sites for glycopeptide antibiotics (Arthur et al., 1994). VanZ decreases the sensitivity of *Enterococcus faecalis* to teicoplanin and oritavancin, but not vancomycin, independent of peptidoglycan modification (Arthur et al., 1995, 1999). The mechanism of VanZ-mediated resistance is not known. In addition to the *vanA* gene cluster, VanZ orthologs not associated with vancomycin resistance gene clusters are present in the genomes of clinically relevant bacteria, such as *Bacillus, Streptococcus, Enterococcus*, and *Clostridium*.

In this work, we compared the ability of *vanZ* from the *vanA* gene cluster encoded on Tn1546, which is present in various strains of *Enterococcus faecium* (*vanZ*_Tei_) (Arthur et al., 1995), and its paralog encoded in the chromosome of *E. faecium* Aus0004 (*vanZ_g_*, locus tag: EFAU004_00030) to confer resistance to glycopeptide antibiotics in *Staphylococcus aureus*, which naturally does not encode *vanZ*. To further confirm the involvement of *vanZ* genes in lipoglycopeptide resistance, we studied the effect of the *vanZ* deletion in *Streptococcus pneumoniae*.

## Materials and Methods

### Strains

*E. faecium* Aus0004, *S. aureus* RN4220, *S. aureus* ATCC29213, *S. pneumoniae* R6, *Escherichia coli* XL1-Blue.

### Antibiotics

Teicoplanin, vancomycin, oritavancin, and chloramphenicol (Sigma-Aldrich, Germany); dalbavancin (MedChemExpress, Sweden); MA79 (Csávás et al., 2015), ERJ390 (Pintér et al., 2009), and SZZS-12 (Szucs et al., 2017); carbenicillin, gentamicin and erythromycin (Duchefa Biochemie, Netherland); vancomycin BODIPY-FL conjugate (Thermo Fisher Scientific, Germany) and fluorescently labeled teicoplanin (Vimberg et al., 2019).

### Preparation of Plasmids Expressing *vanZ*_Tei_ and *vanZ*_g_

The *vanZ*_Tei_ and *vanZ*_g_ genes and their ribosome binding sites were amplified from the plasmid pAT398 (Arthur et al., 1995) and *E. faecium Aus0004* chromosomal DNA, respectively, using the primers TecVanZ_*Sac*I_F, TecVanZ_R, gVanZ_*Sac*I_F, and gVanZ_R (Supplementary Table S1). The PCR products were cloned into the pRMC2 shuttle vector under the control of the anhydrotetracycline (AnhTet)-inducible promoter P*xyl*/*tetO* using *Sac*I and *Eco*RI restriction sites, resulting in the constructs *pRMC2:vanZ_Tei_* and *pRMC2:vanZ_g_*. The constructs were confirmed by sequencing and then electroporated into *S. aureus* RN4220. Using the same procedure, we prepared the constructs *pRMC2:vanZ_Tei_-His* and *pRMC2:vanZ_g_-His* encoding C-terminal His-tagged VanZ variants. However, primers TecVanZhis_R and gVanZhis_R replaced the reverse primers TecVanZ_R and gVanZ_R.

### Construction of the *S. pneumoniae* R6 Δ*vanZ* and *vanZ*-Reverted Strains

Strain Sp539 (Δ*vanZ*) was constructed using a Sweet Janus cassette-based two-step negative selection strategy (Sung et al., 2001; Li et al., 2014). The Sweet Janus cassette contains the kanamycin resistance gene, the recessive *rpsL* gene and the *sacB* gene, which confers sucrose sensitivity (Su^S^), as counterselectable markers. In the first step, 1000 bp fragments corresponding to the upstream and downstream flanking regions of the *vanZ* gene (spr0050) were amplified from the wild-type chromosomal DNA with the primer pairs KB60/KB61 and KB62/KB63, respectively. The Sweet Janus cassette (2807 bp) amplified from the Sweet Janus cassette DNA fragment with the primers DP1/DP2 was attached to the regions flanking *vanZ* by fusion PCR using primers KB60 and KB63. The resulting PCR fragment was used for the transformation of the *S. pneumoniae* R6 strain, and Kan^R^/Su^S^ transformants (Sp537, *vanZ*:*kan sacB*) were selected. The PCR fragments consisting of the upstream and downstream flanking regions of the *vanZ* gene were amplified by the KB60/KB65 and KB64/KB63 primer pairs, respectively, and fused by overlap extension using primers KB60/KB63. The resulting fragment was transformed into the Sp537 strain to obtain Sp539 (Su^R^/Kan^S^). To complement the *vanZ* deletion, we constructed strain Sp635 (Su^R^/Kan^S^; Δ*vanZ*:*vanZ*) by transforming strain Sp537 (Kan^R^/Su^S^) with the PCR fragment amplified with the primers KB60 and KB63 that contained wild-type loci using R6 chromosomal DNA as a template.

### Minimal Inhibitory Concentration (MIC) Measurement

Minimal Inhibitory Concentrations were measured by the broth microdilution method according to ISO standard 20776-1 (EUCAST 2019). *S. aureus* strains with pRMC2, *pRMC2:vanZ_Tei_* and *pRMC2:vanZ_g_* plasmids were cultured in the presence of 25 μg/ml chloramphenicol and 100 ng/ml AnhTet (Sigma-Aldrich, Germany) to induce *vanZ* gene expression. All measurements were performed twice in triplicate. *S. aureus* ATCC29213 was used as a control. MIC values of clinically accepted glycopeptide antibiotics were interpreted according to EUCAST clinical breakpoints (EUCAST, 2019).

### Western Blot Analysis of VanZ Expression

*Staphylococcus aureus* RN4220 strains harboring plasmids *pRMC2:vanZ_Tei_*-His and *pRMC2:vanZ_g_*-His were grown in 2 ml of brain heart infusion medium (Oxoid/Thermo Fisher Scientific, Germany) in the presence of chloramphenicol and AnhTet overnight at 37°C. Cells were harvested and lysed in 1 ml of 1× PBS (phosphate-buffered saline) buffer with 10 μg of lysostaphin (Sigma-Aldrich, Germany) for 15 min at 37°C. Cell debris was removed by centrifugation at 16,000 × *g* for 30 min. The supernatant was then transferred into fresh tubes and centrifuged at 30,000 × *g* for 30 min to separate the membrane and cytosolic fractions. Membrane sediment was resuspended in 50 μl of 1 M urea in 1× PBS. Supernatant proteins were precipitated with 10% TCA, washed twice with ice-cold acetone and resuspended in 50 μl of 1 M urea in 1× PBS buffer. Protein concentration was determined using a bicinchoninic acid (BCA)-based protein estimation kit (Thermo Fisher Scientific, Germany). Proteins were further denatured in SDS-loading buffer at 95°C for 10 min, and 20 μl aliquots were loaded on a 12% SDS-acrylamide gel. After separation by SDS-PAGE, proteins were transferred to a PVDF membrane (Immobilon-P, Merck Millipore, United States) at 15 V for 10 min with a BioRad SemiDry blotting system. His-tagged VanZ was detected with monoclonal anti-His antibody (Sigma-Aldrich, Germany) and subsequently with a secondary goat anti-mouse IgG antibody HRP conjugate (Sigma-Aldrich, Germany). Protein abundance was measured using Immobilon Western HRP Substrate (Merck Millipore, United States), and the signal was developed using the ChemiDoc MP Imaging System (Bio-Rad).

### Binding of Fluorescent Vancomycin and Teicoplanin to *S. aureus* and *S. pneumoniae* Cells

*Staphylococcus aureus* pRMC2, pRMC2:*vanZ*_Tei_ and pRMC2:*vanZ*_g_, *S. pneumoniae* R6, *S. pneumoniae* R6, R6Δ*vanZ*, and R6Δ*vanZ:vanZ* cells were pregrown in Mueller Hinton medium (Oxoid/Thermo Fisher Scientific, Germany) to A_600__nm_ = 0.4. *S. aureus* was pregrown in the presence of chloramphenicol and AnhTet. Cells were harvested by centrifugation and resuspended to A_600__nm_ = 1 in 50 mM Tris–HCl buffer (pH = 7.4). Increasing amounts of Bodipy-Vancomycin (Thermo Fisher Scientific, Germany) or Fluorescent Teicoplanin (Vimberg et al., 2019) were added to 1 ml of resuspended cells. Cells were then incubated for 10 min at room temperature with the fluorescent antibiotics, harvested by centrifugation, washed two times with 50 mM Tris–HCl buffer (pH = 7.4), and finally resuspended in 100 μl of the same buffer. The fluorescence of 70 μl of resuspended cells was measured in automatic gain mode at Ex_490__nm_/Em_520__nm_ in the case of fluorescent vancomycin or Ex_530__nm_/Em_580__nm_ in the case of fluorescent teicoplanin in 96-well black plates (Thermo Fisher Scientific, Germany) by Tecan Infinite 200Pro. The experiment was repeated three times in duplicate.

## Results

### VanZ Reduces the Susceptibility of *S. aureus* and *S. pneumoniae* to Glycopeptide Antibiotics

**FIGURE 1:**
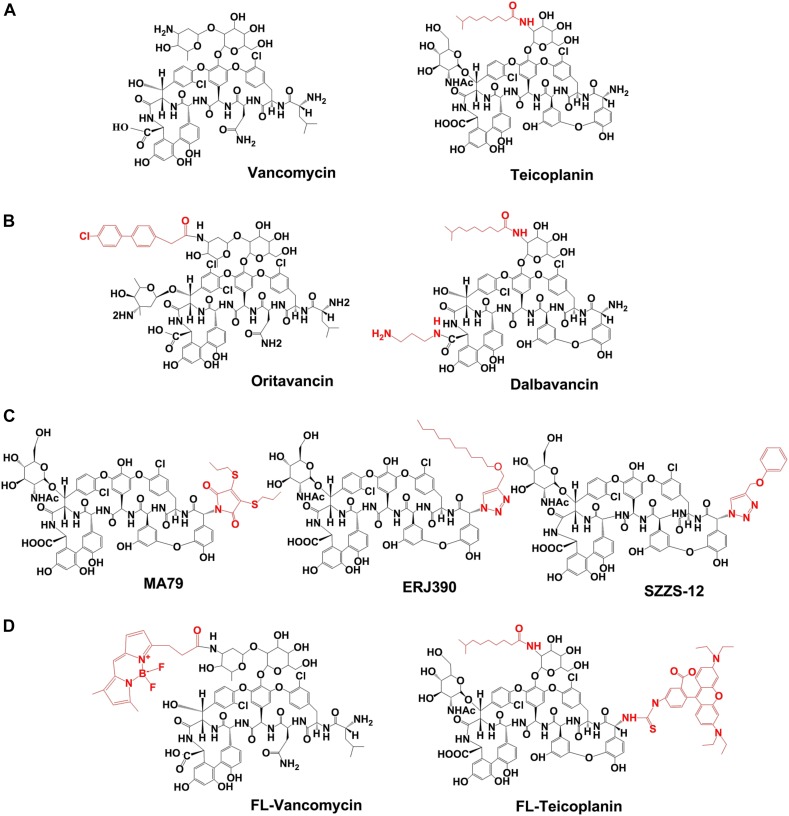
Chemical structures of the glycopeptide antibiotics used in study. **(A)** Natural glycopeptide antibiotics, approved for the clinical use. **(B)** Semisynthetic lipoglycopeptide antibiotics, approved for the clinical use. **(C)** Semisynthetic derivatives of teicoplanin pseudoaglycone. **(D)** Fluorescently labeled vancomycin (FL-Vancomycin) and teicoplanin (FL-Teicoplanin). Lipophilic modifications of the glycopeptide antibiotics are shown in red.

The ability of the *E. faecium vanZ*_Tei_ and *vanZ*_g_ paralogs to confer resistance to glycopeptide antibiotics was tested in *S. aureus*, which naturally does not encode any proteins of the VanZ superfamily. In particular, we determined the susceptibility of *S. aureus* RN4220 expressing *vanZ*_Tei_ and *vanZ*_g_ to the clinically used glycopeptide antibiotic vancomycin (VAN); the lipoglycopeptide antibiotics teicoplanin (TEI), oritavancin (ORI), and dalbavancin (DALB); and three experimental lipoglycopeptide antibiotics derived from teicoplanin pseudoaglycone: MA79 (Csávás et al., 2015), ERJ390 (Pintér et al., 2009) and SZZS-12 (Szucs et al., 2017; Figure 1). In addition, the non-glycopeptide antibiotics carbenicillin (CARB, cell wall-targeting) gentamicin (GEN, 30S ribosome-targeting) and erythromycin (ERY, 50S ribosome-targeting) were used as controls.

As shown in Table 1, the expression of *vanZ*_g_ decreased the susceptibility of *S. aureus* to TEI and ERJ390 16-fold, to DALB four-fold, and to ORI and MA79 two times, and the expression of *vanZ*_g_ had no effect on the susceptibility of *S. aureus* to VAN, SZZS-12 or the control drugs. Similar to *vanZ*_g_, the expression of *vanZ*_Tei_ decreased the susceptibility of *S. aureus* to ERJ390 16-fold but had less or no activity against TEI, ORI and DALB. However, at the same time, cells expressing *vanZ*_Tei_ were more active against MA79 and SZZS-12 (Table 1). To test whether different levels of protein expression cause different activities of VanZg and VanZTei, we performed Western blot analysis of the strains expressing C-terminal His-tagged versions of VanZ proteins. However, the analysis showed that both proteins were expressed at similar levels and that they were localized exclusively in the cell membrane (Supplementary Figure S1).

**TABLE 1:** Summary of the MICs of glycopeptide and non-glycopeptide antibiotics against *S. aureus* RN4220 and *S. pneumoniae* R6, expressing or not expressing VanZ.

**MIC (μg/ml)**	**ATCC 29213**	**RN4220 pRMC2**	**RN4220 *vanZ*_Tei_**	**RN4220 *vanZ*_g_**	**R6**	**R6 Δ*vanZ***	**R6 Δ*vanZ*:*vanZ***
VAN	0.25	0.5	0.5	1	0.25	0.25	0.25
TEI	0.125	0.5	1	**8**	0.125	*0.03125*	0.125
ORI	0.125	0.125	0.125	**0.25**	0.0078	*0.0039*	0.0078
DALB	0.125	0.125	0.125	**0.5**	0.03125	*0.0078*	0.03125
MA79	0.25	0.125	0.5	0.25	0.5	*0.25*	0.5
ERJ390	0.125	0.0156	0.25	0.25	0.5	*0.125*	0.5
SZZS-12	0.125	0.0156	0.0625	0.0156	0.5	*0.125*	0.5
CARB	0.25	0.25	0.25	0.25	0.0625	0.0625	0.0625
GEN	1	1	1	1	0.25	0.25	0.25
ERY	0.125	0.125	0.125	0.125	0.125	0.125	0.125

*VAN, vancomycin; TEI, teicoplanin; ORI, oritavancin; DALB, dalbavancin; CARB, carbenicillin; GEN, gentamicin; ERY, erythromycin. MIC values demonstrating resistance to clinically accepted glycopeptide antibiotics, according to EUCAST (2019) clinical breakpoints, are marked in bold. MIC values of the experimental glycopeptide antibiotics that increased due to *vanZ* expression are underlined. Decreased MIC values against *S. pneumoniae* R6Δ*vanZ* in comparison to wild-type *S. pneumoniae* R6 are in italics.*

To further explore the effect of VanZ proteins on the resistance to glycopeptide antibiotics in the natural genetic background, we employed *S. pneumoniae* R6, which encodes the VanZ ortholog encoded by genome locus spr0050. We constructed a clean knockout of *vanZ* (Δ*vanZ*) and complemented *vanZ* in the *S. pneumoniae* R6 genome and tested the susceptibility of the strains to antibiotics. According to MIC measurements, the Δ*vanZ* mutant was up to four-fold more sensitive to lipoglycopeptide antibiotics, but not to VAN or non-glycopeptide antibiotics, than the wild-type strain (Table 1). Altogether, these data indicate that the transmembrane proteins VanZ_g_ and VanZ_Tei_, as well as VanZ from *S. pneumoniae*, decrease susceptibility to TEI and its derivatives, while they have no or a minor effect on VAN and its derivative ORI.

### VanZ Decreases the Binding of FL-Teicoplanin to *S. aureus* and *S. pneumoniae*

**FIGURE 2:**
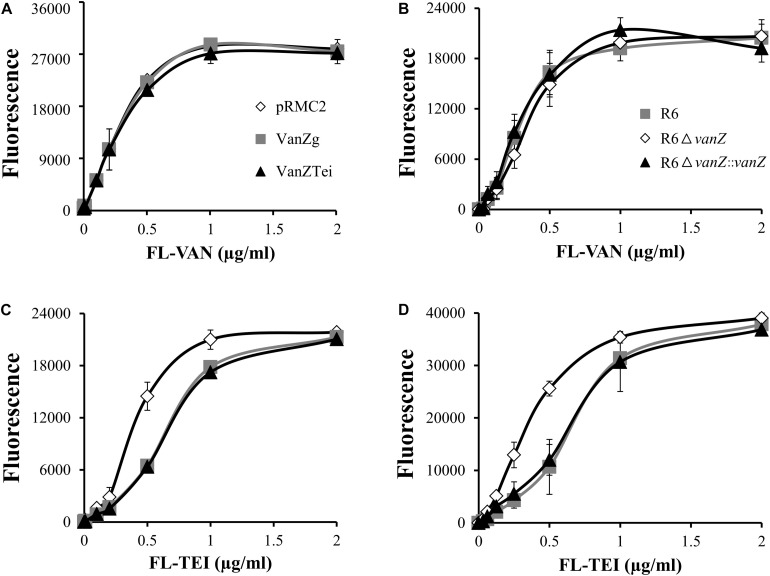
Binding of fluorescent-vancomycin (FL-VAN) and fluorescent-teicoplanin (FL-TEI) to *S. aureus* RN4220, expressing *vanZ*_g_ and *vanZ*_Tei_ **(A,C)** or to *S. pneumoniae* R6, R6Δ*vanZ*, and R6Δ*vanZ*:*vanZ* **(B,D)**. FL-VAN and FL-TEI were titrated to the *S. aureus* and *S. pneumoniae* cells grown to exponential growth phase.

To determine whether the expression of VanZ might interfere with the binding of lipoglycopeptide antibiotics to the bacterial surface, we followed the binding of fluorescently labeled VAN and TEI (FL-VAN and FL-TEI) to *S. aureus* expressing *vanZ*_Tei_ and *vanZ*_g_, as well as to *S. pneumoniae* R6 wild-type, Δ*vanZ* and reverted strains. The titration curves of FL-VAN binding clearly showed that the presence of VanZ did not affected FL-VAN binding to *S. aureus* or *S. pneumoniae* cells (Figures 2A,B). On the other hand, FL-TEI bound less efficiently to *S. aureus* with VanZ_g_ or VanZ_Tei_ than to cells without VanZ (Figure 2C). Similarly, *S. pneumoniae* R6 Δ*vanZ* was saturated with a lower amount of FL-TEI than the wild type (Figure 2D). Altogether, this experiment demonstrates that VanZ proteins might indeed affect the binding of lipoglycopeptide antibiotics to cells.

## Discussion

Here, we showed that orthologous *vanZ* genes from *E. faecium* and *S. pneumoniae* decreased susceptibility to lipoglycopeptide antibiotics independent of their origins, genetic contexts, and host background. Surprisingly, when expressed in *S. aureus*, enterococcal genomic *vanZ*_g_, which is not associated with any glycopeptide resistance gene cluster, was more efficient in conferring resistance to TEI than *vanZ*_Tei_ (Table 1). In addition to *E. faecium* and *S. pneumoniae*, the involvement of genomic *vanZ* genes in the resistance to lipoglycopeptide antibiotics was reported for orthologs from *Streptococcus suis* and *Clostridium difficile* (Lai et al., 2017; Woods et al., 2018; Supplementary Figure S2). In addition to resistance, VanZ proteins might play a more general role in stress response and virulence, as it was observed that the expression of the streptococcal *vanZ* gene was induced by the epithelial antimicrobial peptide LL37 (Lai et al., 2017) and ribosome-targeting antibiotics (Ng et al., 2003) or was essential for lung infection (Hava and Camilli, 2002).

The VanZ-like family (PF04892) comprises a large number of transmembrane proteins of unknown function. Today, more than seven thousand VanZ family representatives can be found in the Pfam protein families database^1^. A phylogenetic tree constructed from 415 VanZ seed sequences, including five VanZ orthologs with activity against glycopeptides, showed that they belong to the same phylogenetic group (Supplementary Figure S3). We hypothesize that all these related VanZ proteins might mediate lipoglycopeptide antibiotic resistance.

Each of the *vanZ* genes conferred various levels of resistance to lipoglycopeptides but did not decrease susceptibility to VAN, and they had only a minor effect on the susceptibility to ORI (Table 1). Correspondingly, *vanZ* expression reduced the binding of FL-TEI but not FL-VAN to the cell surface (Figure 2). The hydrophobic moieties of TEI and DALB are thought to anchor the molecule to the bacterial membrane, thereby improving binding to the lipid II substrate (Beauregard et al., 1995; Kerns et al., 2000; Zeng et al., 2016). On the other hand, the hydrophobic substituent of ORI does not form a membrane anchor; instead, it is an essential part of the secondary binding to pentaglycyl bridge segments of the cell-wall peptidoglycan (Kim et al., 2013, 2017). Thus, VanZ proteins seem to affect the anchoring of lipoglycopeptides to a membrane rather than their binding to the peptidoglycan.

It is of great concern that VanZ orthologs were active against TEI pseudoaglycon derivatives, which represent the newest generation of lipoglycopeptides with promising *in vitro* activity against glycopeptide-resistant strains (Szucs et al., 2017). Similar to ORI, these derivatives show equal competition with FL-TEI and FL-VAN for binding to *S. aureus* cells, and this result correlates with their activity against *vanHAX*-mediated resistance (Vimberg et al., 2019). Nevertheless, whether the hydrophobic substituents interact with a membrane or with the peptidoglycan needs to be determined for these compounds.

The acquisition and spread of *vanZ* genes in *S. aureus* could become a critical problem. The *vanZ*_Tei,_ gene, as a part of the *vanA* gene cluster, is occasionally transferred from enterococci into *S. aureus*, leading to highly vancomycin-resistant strains (VRSA) (Chang et al., 2003; Perichon and Courvalin, 2009; Saadat et al., 2014). Although the incidence of such an event remains low, apparently due to the high fitness cost of *vanHAX-*mediated resistance in *S. aureus* (Foucault et al., 2009), VRSA strains may represent progenitors for the generation of *vanZ*_Tei_-carrying mobile genetic elements.

## Conclusion

In conclusion, our data indicate that VanZ family proteins protect bacteria from lipoglycopeptide antibiotics by affecting their binding to the cell surface. Considering that lipophilization of glycopeptides is an effective way to increase their activity, VanZ superfamily proteins commonly found in the genomes of relevant bacteria as well as the horizontal transfer of *vanZ* to *vanZ*-deficient strains represent a potential threat to the activity of the new generation of glycopeptide antibiotics.

## Data Availability Statement

All datasets generated for this study are included in the article/Supplementary Material.

## Author Contributions

VV designed and performed the MIC measurements, fluorescence, and western-blot experiments. LZ constructed VanZ expressing plasmids and performed MIC measurements. KB constructed vanZ knockout in *S. pneumoniae* and its complementation and contributed to data interpretation and manuscript preparation. PB designed the *S. pneumoniae* experiments, interpreted the data, and contributed to the manuscript preparation. GB designed *S. aureus* experiments, interpreted data, and wrote the manuscript.

## Conflict of Interest

The authors declare that the research was conducted in the absence of any commercial or financial relationships that could be construed as a potential conflict of interest.

## Footnotes

1 http://pfam.xfam.org/family/PF04892#tabview=tab1
