# Supplementary material for: VanZ Reduces the Binding of Lipoglycopeptide Antibiotics to Staphylococcus aureus and Streptococcus pneumoniae Cells
Source: Front Microbiol. 2020 Apr 3;11:566. doi: 10.3389/fmicb.2020.00566 (PMC7146870; doi:10.3389/fmicb.2020.00566)
Supplement: Supplementary file 1 [file Data_Sheet_1.pdf]

Supplementary Material

VanZ Reduces the Binding of Lipoglycopeptide Antibiotics to *Staphylococcus aureus* and *Streptococcus pneumoniae* cells

Vladimir Vimberg[1#], Leona Zieglerová[1#], Karolína Buriánková[2], Pavel Branny[2]* and Gabriela Balíková Novotná[1]*

Supplementary Table 1. List of Primers.

Primer	Sequence	Purpose
TecVanZ_SacI_F	TTTTGAGCTCTAGAGGAAATTATAATGGGAAAAATATT<br>ATCTAGAGGATTGCTAGCTTTATATTTAGTG	$vanZ_{Tei}$ cloning
TecVanZ_R	AAAAGAATTCTTACACGTAATTTATTCTTAAATGGGTACGG<br>TAAACG	$vanZ_{Tei}$ cloning
gVanZ_SacI_F	TTTTGAGCTCGGTTTTCGATGGTATACTTTTTAAATGGAGGA<br>GATGTCTATGCGGTATGTATC	$vanZ_g$ cloning
gVanZ_R	AAAAGAATTCTTATTGAAGACGGACAGTTCGGGTCAAAA<br>ATAAAAAAG	$vanZ_g$ cloning
TecVanZhis_R	AAAAGAATTCTTAATGATGATGATGATGATGTGAACCACCA<br>CCTGGCACGTAATTTATTCTTAAATGGGTACGGTAAACGAG	His tagged $vanZ_{Tei}$ cloning
gVanZhis_R	AAAAGAATTCTTAATGATGATGATGATGATGTGAACCACCA<br>CCTGGTTGAAGACGGAC<br>AGTTCGGGTCAAAAATAAAAAAG	His tagged $vanZ_g$ cloning
KB60/UP_vanZ_F	CTTAAGGAAGTTCTACTTGAG	*S.pneumoniae R6 vanZ* knockout and complementation
KB61/vanZ_sJanusUpR	ATCAAACGGATCGATCCTTAATACAAATACCTCCGTTTGAAG	*S.pneumoniae R6 vanZ* knockout and complementation
KB62/vanZ_sJanusDnF	ACTAAACGTCCAAAAGCATAACTAATGATTAAAAAGGAGAATAT	*S.pneumoniae R6 vanZ* knockout and complementation
KB63/DN_vanZ_R	GAAAACGCCGTGCATCTTCT	*S.pneumoniae R6 vanZ* knockout and complementation
KB64/delta_vanZ_F	CTTCAAACGGAGGTATTTGTACTAATGATTAAAAAGGAGAATAT	*S.pneumoniae R6 vanZ* knockout and complementation
KB65/delta_vanZ_R	ATATTCTCCTTTTTAATCATTAGTACAAATACCTCCGTTTGAAG	*S.pneumoniae R6 vanZ* knockout and complementation
DP1/SweetJanusF	TTAAGGATCGATCCGTTTGATT	*S.pneumoniae R6 vanZ* knockout
DP2/SweetJanusR	TTATGCTTTTGGACGTTTAGTA	*S.pneumoniae R6 vanZ* knockout

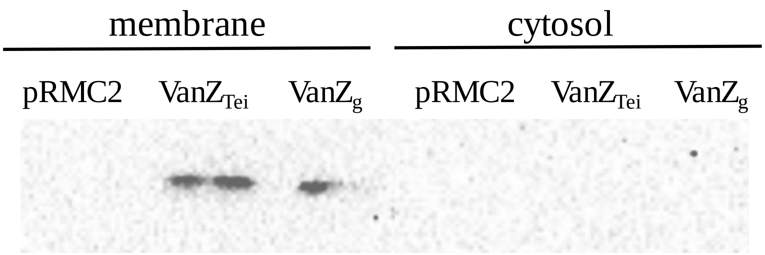

Supplementary Figure 1. Expression and subcellular fractionation of VanZ proteins. A Western blot analysis of *S. aureus* RN4220 with plasmids pRMC2, pRMC2::vanZ$_{Tei}$-His and pRMC2::vanZ$_{g}$-His encoding C-terminal His-tagged VanZ variants. The equivalent amounts of protein fractions were loaded and VanZ proteins were detected using anti-His monoclonal antibody.

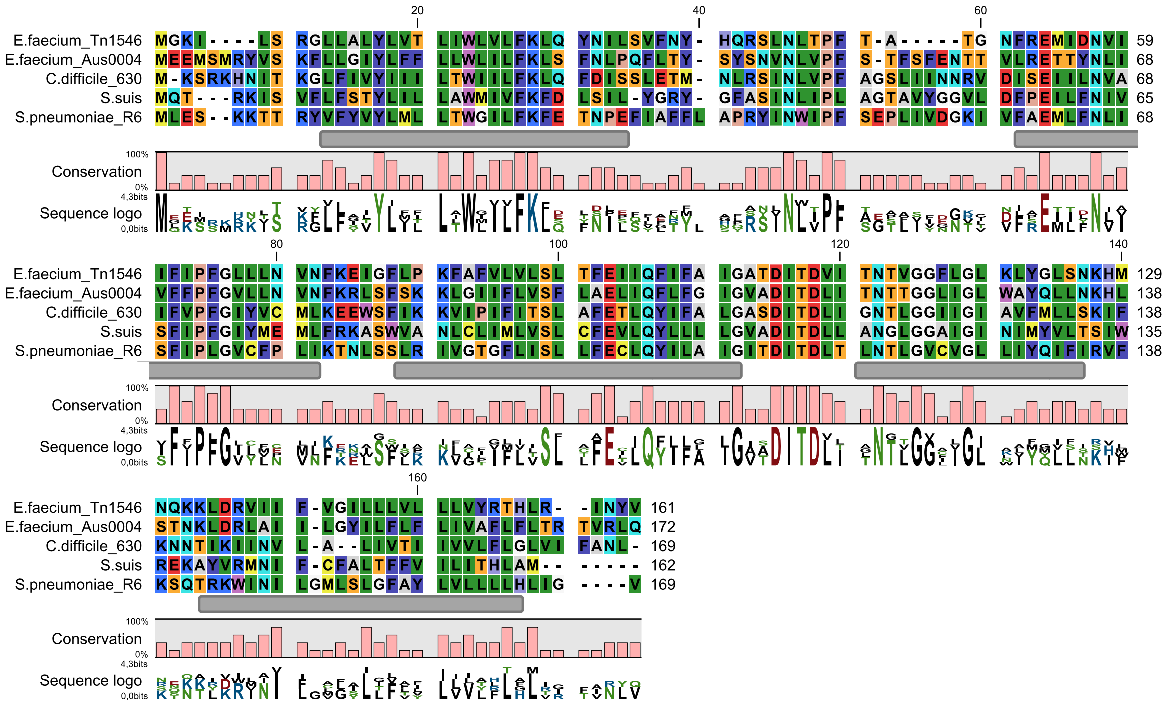

Supplementary Figure 2. The amino acid sequence alignment of the VanZ transmembrane proteins experimentally proven to be involved in lipoglycopeptide resistance. The sequences of VanZ proteins from *E. faecium* Aus0004 (VanZg, WP_002288650), *E. faecium* transposon Tn1546 (VanZTei, WP_000516404), *S. pneumoniae* R6 (WP_000894018), S. *suis* 630 (WP_024419338) and from *C. difficile* (WP_102822090) were aligned using Magellan CLC Main Workbench 6.8.1. Positions of five transmembrane domains predicted by PredictProtein online tool (www.predictprotein.org) are shown by a grey lines under the alignment.

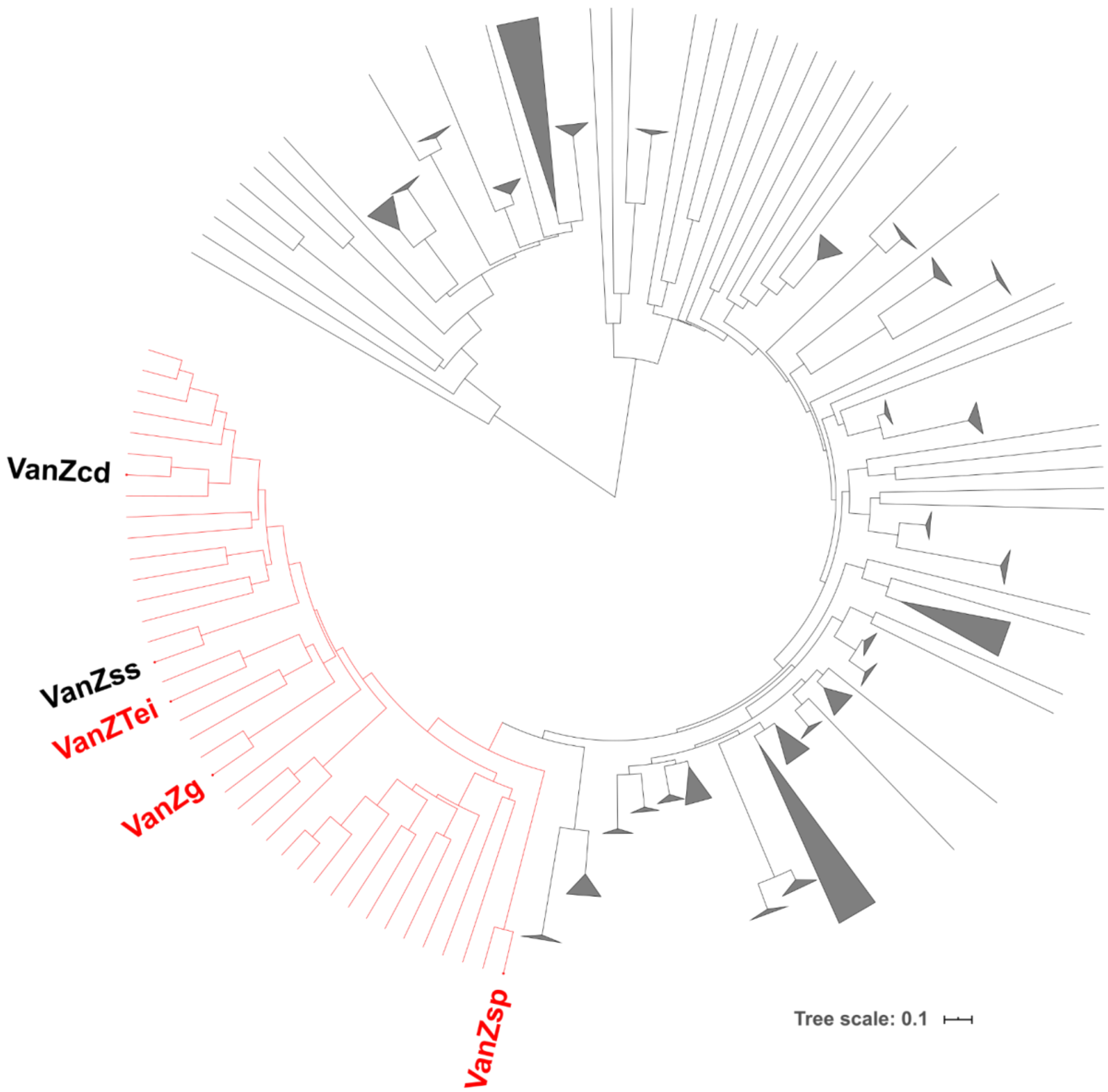

Supplementary Figure 3. VanZ proteins involved in lipoglycopeptide resistance are phylogenetically related. The phylogenetic tree of the VanZ-like family (PF04892) showing relatedness of five characterized VanZ proteins involved in the lipoglycopeptide resistance. VanZ proteins characterized in this study are shown in red. Seed sequences of VanZ proteins (415 sequences) were downloaded from Pfam protein families database (http://pfam.xfam.org/family/PF04892#tabview=tab1). These VanZ amino acid sequences were aligned together with sequences of the five VanZ proteins, characterized to be involved in lipoglycopeptide resistance, VanZg (WP_002288650) VanZTei (WP_000516404), *S. pneumoniae* R6 $VanZ_{sp}$ (WP_000894018), S. *suis* 630 $VanZ_{ss}$ (WP_024419338) and *C. difficile* $VanZ_{cd}$ (WP_102822090) using Magellan CLC Main Workbench 6. 8. 1. The phylogenetic analysis was performed by UPGMA algorithm. The phylogenetic tree was saved in Newick format and visualized in iTOL (Letunic and Bork, 2016). The interactive VanZ-like family tree is accessible at https://itol.embl.de/tree/14723123613181811581085626
